# Tri-ponderal mass index and body mass index in prediction of pediatric metabolic syndrome: the CASPIAN-V study

**DOI:** 10.20945/2359-3997000000206

**Published:** 2020-03-18

**Authors:** Mehri Khoshhali, Motahar Heidari-Beni, Mostafa Qorbani, Mohammad Esmaeil Motlagh, Hasan Ziaodini, Ramin Heshmat, Roya Kelishadi

**Affiliations:** 1 Child Growth and Development Research Center Research Institute for Primordial Prevention of Non-Communicable Disease Isfahan University of Medical Sciences Isfahan Iran Department of Pediatrics, Child Growth and Development Research Center, Research Institute for Primordial Prevention of Non-Communicable Disease, Isfahan University of Medical Sciences, IsfahanIran; 2 Child Growth and Development Research Center Research Institute for Primordial Prevention of Non-Communicable Disease Isfahan University of Medical Sciences Isfahan Iran Department of Pediatrics, Child Growth and Development Research Center, Research Institute for Primordial Prevention of Non-Communicable Disease, Isfahan University of Medical Sciences, Isfahan, Iran; 3 Non-Communicable Diseases Research Center Alborz University of Medical Sciences Karaj Iran Non-Communicable Diseases Research Center, Alborz University of Medical Sciences, Karaj, Iran, Endocrinology and Metabolism Research Center, Endocrinology and Metabolism Clinical Sciences Institute, Tehran University of Medical Sciences, Tehran, Iran; Endocrinology and Metabolism Research Center Endocrinology and Metabolism Clinical Sciences Institute Tehran University of Medical Sciences Tehran Iran; 4 Non-Communicable Diseases Research Center Endocrinology, and Metabolism Population Sciences Institute Tehran University of Medical Sciences Tehran Iran Department of Epidemiology, Non-Communicable Diseases Research Center, Endocrinology, and Metabolism Population Sciences Institute, Tehran University of Medical Sciences, Tehran, Iran; 5 Department of Pediatrics Ahvaz Jundishapur University of Medical Sciences Ahvaz Iran Department of Pediatrics, Ahvaz Jundishapur University of Medical Sciences, Ahvaz, Iran; 6 Health Psychology Research Center Education Ministry Tehran Iran Health Psychology Research Center, Education Ministry, Tehran, Iran; 7 Chronic Diseases Research Center Endocrinology and Metabolism Population Sciences Institute Tehran University of Medical Sciences Tehran Iran Chronic Diseases Research Center, Endocrinology and Metabolism Population Sciences Institute, Tehran University of Medical Sciences, Tehran, Iran; 8 Child Growth and Development Research Center Research Institute for Primordial Prevention of Non-Communicable Disease Isfahan University of Medical Sciences Isfahan Iran Department of Pediatrics, Child Growth and Development Research Center, Research Institute for Primordial Prevention of Non-Communicable Disease, Isfahan University of Medical Sciences, Isfahan, Iran

**Keywords:** Metabolic syndrome, body mass index, tri-ponderal mass index, pediatric

## Abstract

**Objective:**

Body mass index (BMI) and tri-ponderal mass index (TMI) are anthropometric measures to evaluate body adiposity in the various age groups. The present study aims to compare the predictive value of TMI and BMI for metabolic syndrome (Mets) in children and adolescents of both genders.

**Subjects and methods:**

A cross-sectional study conducted on 3731 Iranian children and adolescents aged 7-18 years obtained from the fifth survey of ‘Childhood and Adolescence Surveillance and Prevention of Adult Non-communicable Disease’ (CASPIAN-V) study. The predictive value of BMI and TMI for MetS were determined using Receiver-operator curves. Logistic regression analysis was used to assess the relationship between these indices with MetS.

**Results:**

52.6% of participants were boys. The mean (standard deviations) age for boys and girls were 12.62 (3.02) and 12.25 (3.05) years, respectively. In boys, the area under the curve (AUC) of TMI was greater than BMI for all age groups. AUC of TMI was also greater than BMI for age group of 11-14 years (AUC = 0.74; 95% CI (0.67, 0.81)) in girls. Furthermore, our findings showed that odds ratio of Mets for TMI was greater than BMI in age groups of 11-14 years (OR = 1.33 vs 1.22) and 15-18 years (1.16 vs 1.15) in girls and boys, respectively.

**Conclusion:**

TMI and BMI had moderate predictive value for identifying MetS. However, TMI was a better predictor of MetS than BMI in both genders, especially in age groups of 11-14 and 15-19 years for girls and boys.

## INTRODUCTION

Metabolic syndrome (MetS) is defined as a cluster of cardio metabolic risk factors, including central obesity, glucose intolerance, dyslipidemia and raised blood pressure (1). MetS can occur from early life (2). During last decades, MetS has become a major health problem in children and adolescents; because of various factors including epidemiologic transition, double burden of nutritional disorders, and lifestyle changes. MetS is not limited to developed countries (3).

A systematic review determined the worldwide prevalence rates of MetS ranged between 0 and 19.2% among children and adolescents in 2012 (4). Previous studies indicated that MetS is a common metabolic disorder among Iranian children (5-8). A systematic review reported prevalence rates of MetS in the range of 1-22% in Iran (9).

Obesity and overweight in childhood and adolescence have emerged as one of the most important public health concerns in the 21^st^ century (10). It is important to determine the optimal weight-height indices, which are closely correlated with weight but uncorrelated with height across the growing period (11). Several studies determined that the body mass index (BMI: body weight (kg)/height^2^ (m^2^)) is a preferable index in preschool children and adult (12-14). Furthermore, tri-ponderal mass index (TMI: body weight (kg)/height^3^ (m^3^)) may be applicable for newborns (15). Traditionally, the ponderal index was devised in 1921 by Rohrer and cols. (16). It was originally suggested as a corpulence index and has been used as an indicator of fetal nutrition. Several studies showed that the accuracy of TMI is higher than BMI to determine adiposity in infants (17,18).

The general trend of Power (p) in Weight/Height p by age and gender was specified. However, trends of p are still not clear due to population differences in the studies (14). Recently, Peterson et al showed that TMI estimated body fat percentage more accurately than BMI in adolescents aged 8 to 17 years (19). Another study reported reference values of BMI and TMI for age for healthy non-underweight, non-obese millennial children in the Barcelona longitudinal growth study (1995-2017) (20).

The anthropometric measures are reliable, low cost, non-invasive and can be performed without highly technologic equipment by staff with minimal training (21). Many studies have indicated association between anthropometric measures with MetS and its components in children and adolescents (22-25). However, to our knowledge, there is only one study that explored thresholds of gender-specific TMI and FMI ((fat mass)/height^3^) for the prediction of MetS in population of Colombian children and youth (26). The purposes of this study were (a) to compare predictive ability of TMI and BMI for the prediction of Mets among a large population of Iranian children and adolescents and (b) to determine the optimal cutoff values for BMI and TMI by gender and age groups.

## METHODS AND MATERIALS

### Study design and sample

The data of this nationwide cross-sectional study were collected as a part of the “National survey of school student high risk behaviors” (2014-2015), as the fifth survey of the school-based surveillance system entitled **C**hildhood and **A**dolescence **S**urveillance and **P**revent**I**on of **A**dult **N**on-communicable Disease (CASPIAN-V) study. This school-based nationwide health survey was conducted in 30 provinces in Iran. Details on the study protocol have been explained before (27) and here we report it in brief.

The study population consisted of students aged 7-18 years in primary and secondary schools in urban and rural areas across the country. They were selected using multistage stratified cluster sampling method. Sampling within each province was conducted according to the student’s place of residence (urban or rural) and level of education (primary and secondary) using the proportional to size method and with equal sex ratio. 4200 students were randomly selected for blood sampling.

### Physical measurements

A team of trained health care experts recorded information based on approved check lists; they performed the examinations under standard protocols by using calibrated instruments. Weight was measured on a scale placed on a flat ground to the nearest 0.1 kg while subjects wearing a light cloth, and height were measured without shoes to the nearest 0.1 cm (28). BMI was calculated by dividing weight (kg) to height squared (m^2^) and TMI was calculated dividing weight (kg) to height cubed (m^3^). Waist circumference (WC) was measured using a non-elastic tape at a point midway between the lower border of the rib cage and the iliac crest at the end of normal expiration to the nearest 0.1 cm (29).

Blood pressure was measured in the sitting position on the right arm using a mercury sphygmomanometer with an appropriate cuff size. It was measured 2 times at 5-min intervals; systolic and diastolic pressures were recorded and the average was registered (30).

### Blood sampling

Fasting blood samples were obtained from students after 12–14 h of overnight fast. Fasting blood glucose (FBG), triglycerides (TG), total cholesterol (TC), low-density lipoprotein-cholesterol (LDL-C) and high-density lipoprotein-cholesterol (HDL-C) were measured enzymatically by Hitachi auto148 analyzer (Tokyo, Japan) (31,32).

### MetS components

Subject were classified as having MetS, if they had at least three of the following criteria according to the Adult Treatment Panel III (ATP III) criteria modified for the pediatric age group (33).

1. Abdominal obesity was defined as waist-to-height ratio equal to or more than 0.5 (34).
2. Elevated FBG ≥ 100 mg/dL.
3. High serum TG ≥ 100 mg/dL.
4. Low serum HDL-C < 40 mg/dL (except in boy 15-18 y in whom the cut-off was < 45 mg/dL) (35).
5. Elevated blood pressure was defined as either high systolic blood pressure (SBP) (≥ 90^th^ percentile for age, sex and height) or high diastolic blood pressure (DBP) (≥ 90^th^ percentile for age, sex and height) (30).

### Statistical analysis

The results are represented as mean ± standard deviation (SD). Comparisons between means of anthropometric measures in boys and girls were performed using independent Student t test.

Pearson correlation coefficients were used to determine associations between BMI and TMI with weight and height and also MetS components.

The discriminatory power of BMI, TMI and their z-scores for MetS was calculated by areas under the receiver operating characteristic (ROC) curve. Area under the ROC (AUC) value equal to 1 means perfect accuracy. Furthermore, the optimal cutoff values for BMI, TMI and their z-score were obtained using the Youden index (sensitivity+specificity−1) by gender. The association between TMI, BMI and their z-score with risk of MetS was evaluated using logistic regression model by gender and age. The results of logistic regression are presented as odds ratio (OR) and 95% confidence interval (CI).

The analyses data were performed using statistical software STATA 12.0 (STATA Corp, College Station, Texas, USA). All statistical analysis was performed using survey analysis method. P-valuesless than 0.05 were considered as statistically significant.

## RESULTS

The participation rate was 91.5%. In total, 52.4% of them were boys. The mean (standard deviation) age was 12.62 (3.02) and 12.25 (3.05) years for boys and girls, respectively. Table 1 shows the weight, height, BMI and TMI of participants by age and gender. Mean of weight, height and BMI in boys were more than girls in the age group of 7-10 years (*p* < 0.05). However, in the age group of 11-14 years, girls had significantly greater mean of weight and height than boys (*p* < 0.05). In the age group of 15-18 years, mean of weight and height were significantly greater in boys than girls (*p* < 0.05) whereas mean of BMI and TMI in girls was significantly greater than boys (*p* < 0.05).

**Table 1:** Characteristics of participants by age and gender: the CASPIAN-V Study

Gender	Age group (yr)	Boys		Girls		P
	
		N	Mean ± SD	N	Mean ± SD	
Weight (kg)	7-10	565	28.53 ± 8.25	582	26.85 ± 7.59	< 0.001
	11-14	845	41.07 ± 11.89	809	42.49 ± 12.33	0.019
	15-18	602	56.85 ± 17.85	440	54.93 ± 13.42	0.063
	Total	2012	42.27 ± 17.05	1831	40.45 ± 15.46	0.757
Height (cm)	7-10	565	130.78 ± 10.62	582	128.85 ± 9.82	0.002
	11-14	845	148.56 ± 12.13	809	149.86 ± 11.37	0.029
	15-18	602	164.80 ± 16.74	440	159.86 ± 10.41	< 0.001
	Total	2012	148.44 ± 18.56	1831	145.52 ± 16.12	0.096
BMI (kg/m^2^)	7-10	565	16.54 ± 3.74	582	16.01 ± 3.51	0.015
	11-14	845	18.33 ± 3.64	809	18.62 ± 3.82	0.117
	15-18	602	20.45 ± 4.40	440	21.27 ± 4.15	0.003
	Total	2012	18.46 ± 4.18	1831	18.42 ± 4.28	0.001
TMI (kg/m^3^)	7-10	565	12.74 ± 3.46	582	12.48 ± 3.13	0.189
	11-14	845	12.37 ± 2.57	809	12.43 ± 2.41	0.665
	15-18	602	12.46 ± 2.75	440	13.31 ± 2.49	< 0.001
	Total	2012	12.50 ± 2.90	1831	12.66 ± 2.70	< 0.001

BMI: body mass index; TMI: tri-ponderal mass index.

The correlation coefficients between the indices of BMI and TMI with the weight, height and MetS components are showed in Table 2. For both girls and boys in all age groups, BMI had the greatest correlation with weight. Furthermore, WC and systolic and diastolic pressures had significant correlation with BMI and TMI, with the greatest correlation coefficients for BMI. The correlation between BMI and TMI with TG and LDL were also significant in boys aged 15-18 years.

**Table 2:** Correlation between indices of BMI and TMI with weight, height and components of metabolic syndrome: the CASPIAN-V Study

		Age group (yr)	Weight	Height	WC	SBP	DBP	FBG	TG	TC	HDL	LDL
Boys	BMI	7-10	0.74*	0.02	0.44*	0.21*	0.22*	-0.04	0.03	0.005	0.01	0.02
		11-14	0.82*	0.26*	0.67*	0.26*	0.22*	0.014	-0.02	-0.003	-0.02	-0.01
		15-18	0.84*	0.34*	0.73*	0.28*	0.23*	-0.06	0.10*	0.06	0.01	0.10*
	TMI	7-10	0.41*	-0.36*	0.19*	0.15*	0.16*	-0.04	0.04	0.02	-0.01	0.04
		11-14	0.47*	-0.20*	0.43*	0.16*	0.16*	-0.001	-0.02	0.03	-0.01	0.01
		15-18	0.44*	-0.17*	0.44*	0.12*	0.09*	-0.01	0.12*	0.05	0.02	0.10*
Girls	BMI	7-10	0.77*	0.10*	0.44*	0.27*	0.22*	-0.01	0.02	-0.02	-0.05	0.01
		11-14	0.88*	0.34*	0.65*	0.24*	0.15*	-0.01	0.03	-0.03	-0.08*	0.02
		15-18	0.90*	0.27*	0.74*	0.31*	0.23*	0.003	0.02	0.003	-0.06	0.03
	TMI	7-10	0.47*	-0.26*	0.20*	0.16*	0.15*	-0.01	0.01	-0.03	-0.06	-0.0003
		11-14	0.64*	-0.03	0.47*	0.13*	0.08*	-0.02	0.02	-0.06	-0.06	-0.003
		15-18	0.72*	-0.05	0.64*	0.23*	0.18*	-0.02	-0.0001	0.003	-0.04	0.01

WC: waist circumference; SBP: systolic blood pressure; DBP: diastolic blood pressure; FBG: fasting blood glucose; TG: triglycerides; TC: total cholesterol; HDL: high density lipoprotein; LDL: low density lipoprotein; BMI: body mass index; TMI: tri-ponderal mass index.

* P-value < 0.05.

**Figure 1:**
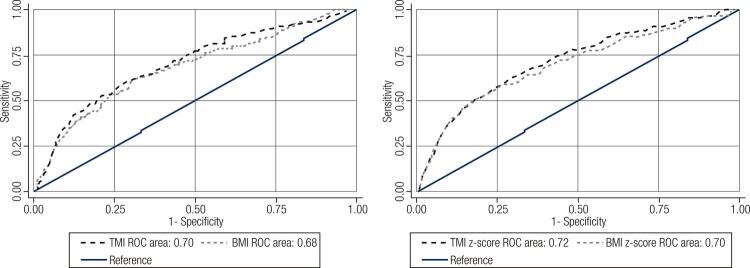
Roc curve of anthropometric indices for predicting metabolic syndrome in children and adolescents: The CASPIAN-V study. BMI: body mass index; TMI: tri-ponderal mass index.

Table 3 shows the optimal cutoffs, corresponding sensitivity and specificity, AUC for BMI and AUC for TMI. Table 4 shows optimal cutoffs, corresponding sensitivity, specificity and AUC of BMI z- score and TMI z-score. In all age groups for boys, TMI and TMI z-score had greater AUC than BMI and BMI z-score for the prediction of Mets. However, the AUC of BMI was greater than TMI for age group of 7-10 years and the AUC of BMI z-score was also greater for groups of 7-10 and 11-15 years in girls. The optimal cutoffs for BMI and TMI in different age groups were obtained using the same method. Figure 1 represents ROC curves of anthropometric indexes for prediction of MetS in the total data.

**Table 3:** ROC curve analysis for indices of BMI and TMI as predictors of metabolic syndrome by gender and age: the CASPIAN-V Study

Gender/Indices	Age groups (yr)	cutoffs	Sensitivity	Specificity	AUC	95% CI
**Boys**						
BMI	7-10	17.71	59.26%	75.67%	0.69	(0.58, 0.80)
	11-14	20.7	60.00%	81.54%	0.74	(0.66, 0.81)
	15-18	23.05	61.76%	81.31%	0.67	(0.55, 0.79)
	Total	19.65	63.21%	69.72%	0.69	(0.63, 0.75)
**T**M**I**	7-10	13.26	66.67%	72.99%	0.73	(0.63, 0.83)
	11-14	12.19	84.44%	58.33%	0.74	(0.67, 0.81)
	15-18	13.26	64.71%	71.69%	0.70	(0.59, 0.80)
	Total	13.17	63.21%	72.75%	0.72	(0.67, 0.78)
**Girls**						
BMI	7-10	18.09	55.56%	84.14%	0.71	(0.60, 0.81)
	11-14	19.73	69.23%	68.68%	0.74	(0.66, 0.81)
	15-18	20.95	76.92%	54.19%	0.64	(0.48, 0.81)
	Total	19.61	60.76%	68.27%	0.67	(0.61, 0.73)
TMI	7-10	14.34	44.44%	87.69%	0.67	(0.56, 0.79)
	11-14	15.35	51.28%	89.92%	0.74	(0.67, 0.81)
	15-18	13.43	76.92%	57.88%	0.64	(0.47, 0.81)
	Total	14.8	45.57%	86.18%	0.68	(0.61, 0.74)

AUC: area under the receiver operating curve; BMI: body mass index; TMI: tri-ponderal mass index.

**Table 4:** ROC curve analysis for indices of BMI z-score and TMI z-score as predictors of metabolic syndrome by gender and age: the CASPIAN-V Study

Gender/Indices	Age groups (yr)	cutoffs	Sensitivity	Specificity	AUC	95% CI
**Boys**						
BMI z- score	7-10	0.88	48.45%	83.46%	0.69	(0.57, 0.80)
	11-14	0.21	70.45%	67.01%	0.73	(0.65, 0.81)
	15-18	0.78	59.38%	83.40%	0.68	(0.56, 0.80)
	Total	0.56	57.43%	78.65%	0.70	(0.65, 0.76)
TMI z- score	7-10	0.42	64.00%	77.82%	0.72	(0.61, 0.83)
	11-14	0.16	70.45%	68.71%	0.74	(0.67, 0.82)
	15-18	0.82	56.25%	82.099%	0.70	(0.60, 0.81)
	Total	0.33	62.38%	72.32%	0.72	(0.67, 0.78)
**Girls**						
BMI z- score	7-10	0.75	55.56%	82.95%	0.71	(0.60, 0.82)
	11-14	0.36	68.42%	70.56%	0.73	(0.64, 0.82)
	15-18	0.16	69.23%	61.39%	0.65	(0.48, 0.81)
	Total	0.68	53.13%	79. 81%	0.71	(0.65, 0.77)
TMI z- score	7-10	0.88	48.15%	84.66%	0.69	(0.58, 0.80)
	11-14	0.61	57.89%	76.80%	0.71	(0.62, 0.80)
	15-18	0.25	69.23%	62.87%	0.65	(0.47, 0.82)
	Total	0.91	46.15%	85.26%	0.69	(0.63, 0.76)

AUC: area under the receiver operating curve; BMI: body mass index; TMI: tri-ponderal mass index.

Table 5 presents the ORs and 95% CI for the association between BMI, TMI and their z-scores with MetS by age and gender. The significant associations were found between BMI and TMI and MetS in girls and boys for all age groups except in girls with age group of 15-18 years. The values of OR for TMI and TMI z-score were greater than BMI and BMI z-score for age groups of 11-14 and 15-18 years in girls and age group of 15-18 years in boys.

**Table 5:** Odds ratio (95% CI) for the association between anthropometric indices with risk of metabolic syndrome by age and gender: The CASPIAN-V study

Age groups (yr)		Boys		Girls	
	
		OR (95% CI)	P	OR (95% CI)	P
7-10	BMI	1.16 (1.08, 1.24)	< 0.001	1.11 (1.04, 1.19)	0.003
	BMI z-score	1.84 (1.40, 2. 41)	< 0.001	1.41 (1.13, 1.76)	0.002
11-14	BMI	1.21 (1.13, 1.29)	< 0.001	1.22 (1.14, 1.32)	< 0.001
	BMI z-score	1.94 (1.51, 2.47)	< 0.001	1.62 (1.36, 1.93)	< 0.001
15-18	BMI	1.15 (1.07, 1.22)	< 0.001	1.11 (0.99, 1.25)	0.071
	BMI z-score	1.89 (1.42, 2.53)	< 0.001	1.32 (0.98, 1.77)	0.065
Total	BMI	1.16 (1.11, 1.20)	< 0.001	1.12 (1.07, 1.17)	0.024
	BMI z-score	1.89 (1.62, 2.20)	< 0.001	1.34 (1.20, 1.50)	0.024
7-10	TMI	1.14 (1.06, 1.21)	< 0.001	1.08 (1.01, 1.16)	0.033
	TMI z-score	1.70 (1.33, 2.19)	< 0.001	1.36 (1.08, 1.71)	0.009
11-14	TMI	1.19 (1.09, 1.29)	< 0.001	1.33 (1.19, 1.49)	< 0.001
	TMI z-score	1.65 (1.32, 2.06)	< 0.001	2.00 (1.52, 2.62)	< 0.001
15-18	TMI	1.16 (1.06, 1.28)	0.002	1.18 (0.97, 1.43)	0.102
	TMI z-score	1.54 (1.19, 2.00)	0.001	1.53 (0.94, 2.49)	0.102
Total	TMI	1.15 (1.10, 1.21)	< 0.001	1.14 (1.08, 1.21)	< 0.001
	TMI z-score	1.63 (1.42, 1.88)	< 0.001	1.58 (1.34, 1.87)	< 0.001

OR: odds ratio; CI: confidence interval; BMI: body mass index; TMI: tri-ponderal mass index.

## DISCUSSION

Previous studies assessed and compared BMI and TMI on adiposity (13) and body fat (19). To the best of our knowledge, this is the first study comparing the accuracy of TMI and BMI indices for Mets in pediatric population. This study indicated that the predictive power of TMI was higher than BMI in the prediction of Mets in boys for all age groups and in the age group of 11-14 years for girls. The optimal cutoffs for BMI (BMI z-score) and TMI (TMI z-score) for predicting MetS were 19.65 (0.56) and 13.17 (0.33) in boys and 19.61 (0.68) and 14.8 (0.91) in girls, respectively. Furthermore, our findings showed that in the age group of 7-10 years, the risk of Mets for BMI (BMI z-score) was higher than TMI (TMI z-score) in both girls and boys; in the age group of 11-14 years, TMI (TMI z-score) was greater than BMI (BMI z-score) in girls; and in age group of 15-18 years, TMI was greater than BMI in both girls and boys but in girls was not statistically significant.

According to findings, weight spurt during puberty spurts is more than twice the height spurt in percentage terms (13). The findings of previous studies showed that TMI is a better adiposity index than BMI during puberty (36), which was similar to the findings of the present study.

Recently, Peterson and cols. showed that TMI estimated body fat levels more accurately than BMI in non-Hispanic white adolescents aged 8 to 17 years. Furthermore, they indicated that TMI could diagnose overweight adolescents more accurately than BMI *z* scores (19). Several studies have found the associations between fat distribution (BF% and /or FMI) and MetS components (37,38). Our findings were concordant with results of these studies.

There are differences between the predictive values of BMI and TMI for MetS in various populations. A cross-sectional study on Brazilian children from public and private schools showed that BMI had AUC at 0.754 in 5th-grade girl students (25). Another cross-sectional study reported that BMI had high predictive value (AUC = 0.98) for MetS among Spanish adolescents of both genders (24). In study on Colombian children and adolescents, the ROC analysis showed that TMI had a moderate discriminatory power to detect MetS (all AUCs for age groups of 9-12 and 13-17 years in both girls and boys were less than 0.755) (26). In the present study, TMI and BMI had moderate diagnostic accuracy for identifying MetS among Iranian children and adolescents. This discrepancy in results could be described by the heterogeneity of the sample populations and or different definitions of pediatric Mets (35).

Previous studies have identified strong effects of puberty on distributions of adiposity (39) and the Mets (40). The present study also showed relationship between BMI and TMI with risk of Mets in puberty age particularly for TMI.

Findings of the present study provide optimal cutoffs for BMI and TMI by age and gender. More studies are suggested in different population (41). The optimal BMI cutoff values to predict MetS in our study were 19.65 kg/m^2^ for boys and 19.61 kg/m^2^ for girls, which differs from values in Spanish adolescents (cutoff = 27 for both gender) (24). Moreover, the optimal TMI cutoff values to predict MetS in Colombian children and adolescents for age groups of 9-12 and 13-17 years were 12.10 and 11.9 years for boys and 12.13 and 12.48 for girls, respectively. However, TMI cutoff values in the present study were greater than values in previous studies for all age groups in both boys and girls. The differences in these values could be due to differences in lifestyle between various population (41). In the current study, the optimal cutoff values for BMI z-score and TMI z-score to predict MetS were less than the values to predict overweight or obesity. This finding maybe because of the high prevalence of high TG and low HDL cholesterol levels among normal-weight children in Iran (42). Moreover, several studies reported CVD risk factors and metabolic syndrome among Asian adults, children and adolescents with normal weight (42-44).

The main limitation of the present study is cross-sectional nature, therefore the cause–effect relationships cannot be inferred from these findings. Another limitation is the lack of information on puberty age in adolescents. The main strengths of our study are its novelty in the pediatric age group and considering a large population-based sample.

In conclusion, the results of present study showed that both BMI and TMI had moderate diagnostic accuracy for identifying MetS in children and adolescents. However, generally TMI was a better predictor of MetS than BMI in both genders. TMI can be considered as an appropriate anthropometric index for large population-based studies in the pediatric age group.

Ethics approval and consent to participate: study protocols were reviewed and approved by ethical committees and other relevant national regulatory organizations. The Research and Ethics council of Isfahan University of Medical Sciences approved the study (Project number: 194049). After complete explanation of the study objectives and protocols, written informed consent and verbal consent were obtained from the parents and students, respectively.

## References

[1] Wu Y-E, Zhang C-L, Zhen Q. Metabolic syndrome in children (Review). Exp Ther Med. 2016;12:2390-4. 10.3892/etm.2016.3632 PMC5038558 27698739

[2] Kelishadi R, Poursafa P. A review on the genetic, environmental, and lifestyle aspects of the early-life origins of cardiovascular disease. Curr Probl Pediatr Adolesc Health Care. 2014;44:54-72. 10.1016/j.cppeds.2013.12.005 24607261

[3] Kelishadi R. Childhood overweight, obesity, and the metabolic syndrome in developing countries. Epidemiol Rev. 2007;29:62-76. 10.1093/epirev/mxm003 17478440

[4] Friend A, Craig L, Turner S. The Prevalence of Metabolic Syndrome in Children: A Systematic Review of the Literature. Metab Syndr Relat Disord. 2013;11:71-80. 10.1089/met.2012.0122 23249214

[5] Khashayar P, Heshmat R, Qorbani M, Motlagh ME, Aminaee T, Ardalan G, et al. Metabolic Syndrome and Cardiovascular Risk Factors in a National Sample of Adolescent Population in the Middle East and North Africa : The CASPIAN III Study. Int J Endocrinol. 2013:1-8. 10.1155/2013/702095 PMC3580930 23476647

[6] Qorbani M, Mehrkash M, Kelishadi R, Mohammadian S, Mousavinasab F, Esmaeil M, et al. Obesity and Metabolic Syndrome Among a Representative Sample of Iranian Adolescents. Southeast Asian J Trop Med Public Health. 2012;43:756-63. 23077856

[7] Mirhosseini N-Z, Yusoff NAM, Shahar S, Parizadeh SMR, Mobarhen MG, Shakery MT. Prevalence of the metabolic syndrome and its influencing factors among adolescent girls in Mashhad, Iran. Asia Pac J Clin Nutr. 2009;18:131-6. 19329406

[8] Ostovaneh MR, Zamani F, Sharafkhah M, Ansari-Moghaddam A, Akhavan Khaleghi N, Saeedian FS, et al. Prevalence of metabolic syndrome in Amol and Zahedan, Iran: A population based study. Arch Iran Med. 2014;17:477-82. 24979559

[9] Kelishadi R, Hovsepian S, Djalalinia S, Jamshidi F, Qorbani M. A systematic review on the prevalence of metabolic syndrome in Iranian children and adolescents. J Res Med Sci. 2016;21:1-9. 10.4103/1735-1995.192506 PMC5244691 28163736

[10] Abarca-Gómez L, Abdeen ZA, Hamid ZA, Abu-Rmeileh NM, Acosta-Cazares B, Acuin C, et al. Worldwide trends in body-mass index, underweight, overweight, and obesity from 1975 to 2016: a pooled analysis of 2416 population-based measurement studies in 1289 million children, adolescents, and adults. Lancet. 2017;390:2627-42. 10.1016/S0140-6736(17)32129-3 PMC5735219 29029897

[11] NCD Risk Factor Collaboration (NCD-RisC). A century of trends in adult human height. Elife. 2016;26;5:1-29. 10.7554/eLife.13410 PMC4961475 27458798

[12] Hattori K. Age Change of Allometry. J Anthr Soc Nippon. 1975;83:29-38.

[13] Cole TJ. Weight/heightp compared to weight/height2 for assessing adiposity in childhood: influence of age and bone age on p during puberty. Ann Hum Biol. 1986;13:433-51. 10.1080/03014468600008621 3800308

[14] Hattori K, Hirohara T. Age change of power in weight/heightp indices used as indicators of adiposity in Japanese. Am J Hum Biol. 2002;14:275-9. 10.1002/ajhb.10037 11891939

[15] Lehingue Y, Remontet L, Munoz F, Mamelle N. Birth ponderal index and body mass index reference curves in a large population. Am J Hum Biol. 1998;10:327-40. 10.1002/(SICI)1520-6300(1998)10:3<327::AID-AJHB8>3.0.CO;2-F 28561395

[16] Cooley SM, Donnelly JC, Walsh T, Kirkham C, Gillan J, Geary MP. Ponderal index (PI) vs birth weight centiles in the low-risk primigravid population: which is the better predictor of fetal wellbeing? J Obstet Gynaecol. 2012;32:439-43. 10.3109/01443615.2012.667172 22663314

[17] Freedman DS, Wang J, Thornton JC, Mei Z, Sopher AB, Pierson RN, et al. Classification of body fatness by body mass index-for-age categories among children. Arch Pediatr Adolesc Med. 2009;163:805-11. 10.1001/archpediatrics.2009.104 PMC2846460 19736333

[18] Howe LD, Tilling K, Benfield L, Logue J, Sattar N, Ness AR, et al. Changes in ponderal index and body mass index across childhood and their associations with fat mass and cardiovascular risk factors at age 15. Hernandez AV, editor. PLoS One. 2010;5:1-13. 10.1371/journal.pone.0015186 PMC2999567 21170348

[19] Peterson CM, Su H, Thomas DM, Heo M, Golnabi AH, Pietrobelli A, et al. Tri-Ponderal Mass Index vs Body Mass Index in Estimating Body Fat During Adolescence. JAMA Pediatr. 2017;35294:1-8. 10.1001/jamapediatrics.2017.0460 PMC5710345 28505241

[20] Carrascosa A, Yeste D, Moreno-Galdó A, Gussinyé M, Ferrández A, Clemente M, et al. Índice de masa corporal e índice de masa triponderal de 1.453 niños no obesos ni malnutridos de la generación del milenio. Estudio longitudinal de Barcelona. An Pediatría. 2018. 89(3):137-43. 10.1016/j.anpedi.2017.12.016 29478880

[21] Vogt BP, Ponce D, Caramori JCT. Anthropometric Indicators Predict Metabolic Syndrome Diagnosis in Maintenance Hemodialysis Patients. Nutr Clin Pract. 2016;31:368-74. 10.1177/0884533615601849 26341917

[22] Wicklow BA, Becker A, Chateau D, Palmer K, Kozyrskij A, Sellers EAC. Comparison of anthropometric measurements in children to predict metabolic syndrome in adolescence: Analysis of prospective cohort data. Int J Obes. 2015;39:1070-8. 10.1038/ijo.2015.55 25869598

[23] Kelishadi R, Heidari-Beni M, Qorbani M, Motamed-Gorji N, Motlagh ME, Ziaodini H, et al. Association between neck and wrist circumferences and cardiometabolic risk in children and adolescents: The CASPIAN-V study. Nutrition. 2017;43:32-8. 10.1016/j.nut.2017.06.009 28935142

[24] Perona JS, Schmidt-RioValle J, Rueda-Medina B, Correa-Rodríguez M, González-Jiménez E. Waist circumference shows the highest predictive value for metabolic syndrome, and waist-to-hip ratio for its components, in Spanish adolescents. Nutr Res. 2017;45:38-45. 10.1016/j.nutres.2017.06.007 29037330

[25] Andaki ACR, Tinôco ALA, Mendes EL, Andaki Júnior R, Hills AP, Amorim PRS. Anthropometry and physical activity level in the prediction of metabolic syndrome in children. Public Health Nutr. 2014;17: 2287-94. 10.1017/S136898001300253X PMC10282609 24063585

[26] Ramírez-Vélez R, Correa-Bautista JE, Carrillo HA, González-Jiménez E, Schmidt-RioValle J, Correa-Rodríguez M, et al. Tri-Ponderal Mass Index vs. Fat Mass/Height3 as a Screening Tool for Metabolic Syndrome Prediction in Colombian Children and Young People. Nutrients. 2018;10(4). pii: E412. 10.3390/nu10040412 PMC5946197 29584641

[27] Motlagh M, Ziaodini H, Qorbani M, Taheri M, Aminaei T, Goodarzi A, et al. Methodology and early findings of the fifth survey of childhood and adolescence surveillance and prevention of adult noncommunicable disease: The caspian-v study. Int J Prev Med. 2017;8:1-9. 10.4103/2008-7802.198915 PMC5288959 28217266

[28] WHO. Physical status: the use and interpretation of anthropometry. Report of a WHO Expert Committee. World Health Organ Tech Rep Ser. 1995;854:1-452. 8594834

[29] Knowles KM, Paiva LL, Sanchez SE, Revilla L, Lopez T, Yasuda MB, et al. Waist Circumference, Body Mass Index, and Other Measures of Adiposity in Predicting Cardiovascular Disease Risk Factors among Peruvian Adults. Int J Hypertens. 2011;2011:931402. 10.4061/2011/931402 PMC3034939 21331161

[30] Adolescents N. The Fourth Report on the Diagnosis, Evaluation, and Treatment of High Blood Pressure in Children and Adolescents. Pediatrics. 2004;114:555-76. 15286277

[31] Friedewald WT, Levy RI, Fredrickson DS. Estimation of the concentration of low-density lipoprotein cholesterol in plasma, without use of the preparative ultracentrifuge. Clin Chem. 1972;18:499-502. 4337382

[32] McNamara JR, Schaefer EJ. Automated enzymatic standardized lipid analyses for plasma and lipoprotein fractions. Clin Chim Acta. 1987;166:1-8. 10.1016/0009-8981(87)90188-4 3608193

[33] National Cholesterol Education Program (NCEP) Expert Panel on Detection. Evaluation, and Treatment of High Blood Cholesterol in Adults (Adult Treatment Panel III). Third Report of the National Cholesterol Education Program (NCEP) Expert Panel on Detection, Evaluation, and Treatment of High Blood Cholesterol in Adults (Adult Treatment Panel III) final report. Circulation. 2002;106:3143-421. 12485966

[34] Li C, Ford ES, Mokdad AH, Cook S. Recent Trends in Waist Circumference and Waist-Height Ratio Among US Children and Adolescents. Pediatrics. 2006;118:1390-8. 10.1542/peds.2006-1062 17079540

[35] Zimmet P, Alberti KGM, Kaufman F, Tajima N, Silink M, Arslanian S, et al. The metabolic syndrome in children and adolescents – an IDF consensus report. Pediatr Diabetes. 2007;8:299-306. 10.1111/j.1399-5448.2007.00271.x 17850473

[36] Cole TJ. A method for assessing age-standardized weight-for-height in children seen cross-sectionally. Ann Hum Biol. 1979;6:249-68. 10.1080/03014467900007252 496386

[37] McCarthy HD. Body fat measurements in children as predictors for the metabolic syndrome: focus on waist circumference. Proc Nutr Soc. 2006;65:385-92. 10.1017/s0029665106005143 17181905

[38] Ramírez-Vélez R, Correa-Bautista JE, Sanders-Tordecilla A, Ojeda-Pardo ML, Cobo-Mejía EA, Castellanos-Vega RDP, et al. Percentage of body fat and fat mass index as a screening tool for metabolic syndrome prediction in Colombian university students. Nutrients. 2017;9(9). pii: E1009. 10.3390/nu9091009 PMC5622769 28902162

[39] Loomba-Albrecht LA, Styne DM. Effect of puberty on body composition. Curr Opin Endocrinol Diabetes Obes. 2009;16:10-5. 10.1097/med.0b013e328320d54c 19115520

[40] Reinehr T, Wolters B, Knop C, Lass N, Holl RW. Strong effect of pubertal status on metabolic health in obese children: a longitudinal study. J Clin Endocrinol Metab. 2015;100:301-8. 10.1210/jc.2014-2674 25243573

[41] Chen X, Liu Y, Sun X, Yin Z, Li H, Deng K, et al. Comparison of body mass index, waist circumference, conicity index, and waist-to-height ratio for predicting incidence of hypertension: the rural Chinese cohort study. J Hum Hypertens. 2018;32(3):228-35. 10.1038/s41371-018-0033-6 29416119

[42] Kelishadi R, Cook SR, Motlagh ME, Gouya MM, Ardalan G, Motaghian M, et al. Metabolically Obese Normal Weight and Phenotypically Obese Metabolically Normal Youths: The CASPIAN Study. J Am Diet Assoc. 2008;108:82-90. 10.1016/j.jada.2007.10.013 18155992

[43] Li YP, Yang XG, Zhai FY, Piao JH, Zhao WH, Zhang J, et al. Disease risks of childhood obesity in China. Biomed Environ Sci. 2005;18:401-10. 16544522

[44] Vikram NK, Pandey RM, Misra A, Sharma R, Devi JR, Khanna N. Non-obese (body mass index < 25 kg/m^2^) Asian Indians with normal waist circumference have high cardiovascular risk. Nutrition. 2003;19:503-9. 10.1016/s0899-9007(02)01083-3 12781849

